# Magnetoferritin: Process, Prospects, and Their Biomedical Applications

**DOI:** 10.3390/ijms20102426

**Published:** 2019-05-16

**Authors:** Le Xue, Dawei Deng, Jianfei Sun

**Affiliations:** 1 Department of Biomedical Engineering, School of Engineering, China Pharmaceutical University, Nanjing 210009, China; jsxuele@163.com; 2 State Key Laboratory of Bioelectronics, School of Biological Science and Medical Engineering, Southeast University, Nanjing 210096, China

**Keywords:** ferritin, magnetoferritin, magnetic protein, tumor targeting, biomedical applications

## Abstract

Ferritin is a spherical iron storage protein composed of 24 subunits and an iron core. Using biomimetic mineralization, magnetic iron oxide can be synthesized in the cavity of ferritin to form magnetoferritin (MFt). MFt, also known as a superparamagnetic protein, is a novel magnetic nanomaterial with good biocompatibility and flexibility for biomedical applications. Recently, it has been demonstrated that MFt had tumor targetability and a peroxidase-like catalytic activity. Thus, MFt, with its many unique properties, provides a powerful platform for tumor diagnosis and therapy. In this review, we discuss the biomimetic synthesis and biomedical applications of MFt.

## 1. Introduction of Ferritin and Magnetoferritin

**Figure 1:**
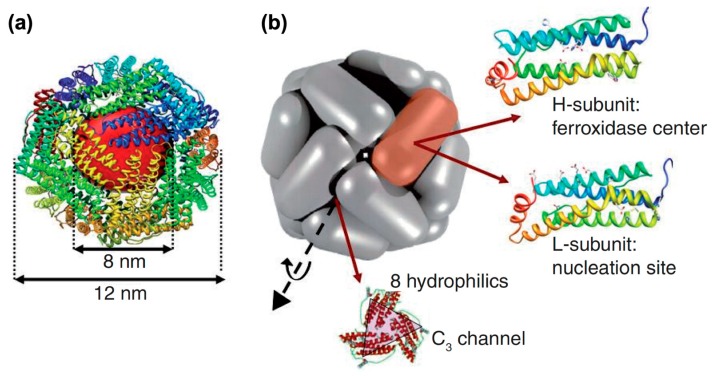
The structure of native ferritin: (**a**) The spherical cage and iron core of ferritin; (**b**) ferritin is composed of H chain subunits and L chain subunits, with 8 hydrophilic channels, which formed a 3-fold axes. Reprinted with permission from Ref 7. Copyright 2010 Newlands Press Ltd.

Ferritin belongs to a family of iron storage proteins, which was first isolated from horse spleen in 1937 by Laufberger [1]. Thereafter, ferritin was widely discovered in bacteria, fungi, plants, humans, and other mammals [1,2,3]. While amino acid sequences varied among species, a few key structural residues of the iron-binding motifs were highly conservative or conservatively substituted [4], thereby leading to an essentially similar architecture and structural feature among ferritins [5]. The typical structure of ferritin involves a protein shell and an iron core [2]. In general, 24 subunits form a spherical cage (outer diameter 12 nm, inner diameter 8nm) as the protein shell, whereas the iron core was formed in the protein cavity with a type of ferrihydrite (5Fe_2_O_3_·9H_2_O) (Figure 1) [5,6,7]. The ferrihydrite of natural ferritin is antiferromagnetic [8], however, the inorganic core of ferritin could be reconstituted with magnetic iron core (magnetite or maghemite) in vitro to form an artificial magnetic protein, which was termed as magnetoferritin (MFt) [2].

In humans, the shell of ferritin is composed of two types of subunits, an H chain of 21 kDa and an L chain of 19 kDa [2]. H and L stand for heavy and light, furthermore, the H chain was named for its discovery in mammalian heart, whereas the L chain was named for its discovery in mammalian liver [9]. The highly conserved di-iron binding site in the H chain binds and oxidizes ferrous ions, and this intrasubunit ferroxidase center does not exist in the L chain [10]. When the H chain catalyzes the oxidation of Fe (II) to Fe (III) with low solubility, spontaneous nucleation will occur, and result in the formation of the ferrihydrite core inside the cavity [11].

According to the distribution within the human body, ferritin is divided into intracellular and extracellular ferritin [1]. In most tissues, ferritins are mainly located in cytosol with a minor fraction in nucleus and a different type in mitochondria. In addition, ferritins are involved in iron storage and iron homeostasis in vivo. Ferritin can also protect mitochondria and DNA from oxidative damage and iron toxicity [1,12]. The heavy subunit of ferritin plays a role in cell proliferation, angiogenesis, and stem cell expansion [13]. It has previously been reported that the ferritin heavy chain is involved in controlling cancer cell growth [14]. Recently, it was demonstrated that the ferritin heavy chain impacted cell viability by regulating the expression of miRNAs [13,15,16]. In addition, oncogenic miRNAs can change the expression of the ferritin heavy chain and its pseudogenes to develop cancer [17]. The expression of ferritin in tumor cells could also impact chemoresistance in tumor therapy [18]. In addition, Cytosolic ferritin is involved in neurodegenerative disorders [19]. Changes in the extracellular ferritin concentration are associated with a variety of diseases [1]. For example, it was found that the level of serum ferritin is associated with anemia [20], liver disease [21], and cancer [22,23]. Furthermore, serum levels of ferritin were able to predict liver fibrosis and cirrhosis [24]. The serum ferritin concentration could also serve as a reference in breast cancer diagnosis [25]. Moreover, it has been found that serum ferritin and colorectal cancer were significantly correlated [26]. While the biological function and source of serum ferritin remains to be elucidated [27], these data indicated that ferritin might play an important role in tumor formation.

The natural structure of ferritin provides an appropriate template for the synthesis of nanoparticles. The iron core of nature ferritin can be removed by reduction, and inorganic clusters or nanoparticles can be synthesized in the cavity by mineralization [28]. The mineral core, semiconductor core, and metal/metal alloy core were all introduced to the modification or reformation of ferritin, such as Mn_3_O_4_ [29], Co_3_O_4_ [30], CaCO_3_ [31], CdSe [32], ZnSe [33], Au [34], and CoPt [29], which could be synthesized with a unified shape and size distribution. 

Magnetite nanoparticles were found inside pathological tissues involving neurodegenerative diseases [35]. The core of native ferritin might turn from ferrihydrite to magnetite, thereby forming biogenic magnetoferritin [19]. This might be the result from iron detoxification of ferritins by oxidizing toxic Fe (II) [36]. Similarly in vitro, Magnetic iron oxide nanoparticles (Fe_3_O_4_, γ-Fe_2_O_3_) can be synthesized into the empty shell of ferritin to form a novel biocompatible material [28]. And we call this nanomaterial with ferritin shell and magnetic iron oxide core as magnetoferritin, MFt. The ferritin provides a good template for the synthesis of uniform size nanoparticles, and the protein shell also enable MFts non-interacting and well-dispersed [37]. MFt with an average size of 12 nm is generally superparamagnetic due to its magnetic iron oxide core. In addition, MFt showed low toxicity and excellent biocompatibility [38,39]. Subsequently, the synthesis and application of MFt was greatly expanded [2,40,41]. Genic recombinant ferritins composed of H chain ferritin (HFt) were biologically synthesized with better properties for biomedical applications. Recently, it was found that MFt composed of only H Chains (MHFt) has the ability to target tumor cells and has a peroxidase-like activity [41]. Transferrin receptor 1 (TfR1) is a typical type II transmembrane glycoprotein, which forms a homodimer on the surface of the cell membrane [42]. It has been reported that HFt itself specifically binds to human cells via interacting with TfR1 [43]. After binding to TfR1 on the cell surface, H-ferritin is transported into intracellular bodies and lysosomes. Because tumor cells overexpress TfR1 [44], HFt nanoparticles are expected to be a potential candidate for tumor targeting with high biocompatibility and safety. This means that MHFt can target tumor cells without targeting ligand functionalization. 

With its unique structural future, MFt exhibits stability, biocompatibility, and the capability to mineralize magnetic core inside the cage. MFt generally showed superparamagnetic behavior and magnetic anisotropy, which could be used in MRI and hyperthermia [45,46,47]. The shell of MFt can be functionalized by chemical and genetic engineering [2]. In addition, mineralization within the cavity can be modified to gain ideal properties. MFt provides a powerful nanoplatform for biomedical applications [1]. This biomimetic ferritin with superparamagnetic Fe_3_O_4_ or γ-Fe_2_O_3_ core [2,28,48], MFt, has attracted worldwide attention. Here, an extensive overview is presented on the synthesis, properties, modifications, and biomedical applications of MFt.

## 2. Synthesis of Magnetoferritin

Mann et al. firstly reported the synthesis of the magnetic protein MFt [28]. The magnetic mineral core could be reconstituted in the nano cavity of horse spleen ferritin by utilizing the unusual stability of the ferritin at high temperatures and pH. Firstly, apoferritin (apoFt) was prepared by removing iron from native ferritin. Then, at 60 °C and pH 8.5, an Fe(II) solution was slowly added to an apoferritin solution with slow oxidation by air. Electron diffraction patterns cannot identify the crystals formed in the protein cavity as ferrimagnetic minerals, magnetite or maghemite. However, considering anaerobic reaction conditions and black mineral core, the most likely possibility seems to be magnetite (Fe_3_O_4_). Next, an improved synthesis used trimethylamine-*N*-oxide as an oxidant to obtain MFt, containing ferrimagnetic nanocrystals of various iron loadings (from 100 to 3150 Fe atoms/protein molecule) [49]. In this research, studies on magnetic properties suggested that the mineral cores of MFt synthesized above should be maghemite (γ-Fe_2_O_3_), rather than magnetite (Fe_3_O_4_). All MFt samples were superparamagnetic above 100 K. Samples with theoretical loadings more than 1000 Fe atoms exhibited greater dimensions than the protein cavity. When iron loadings increased, the superparamagnetic blocking temperature of the MFt also increased from 10 to 100 K. However, the initial core of horse spleen ferritin needed to be removed before reconstituting the magnetic mineral core, which was harmful for the shell of ferritin, and resulted in the aggregation of ferritin [28].

**Figure 2:**
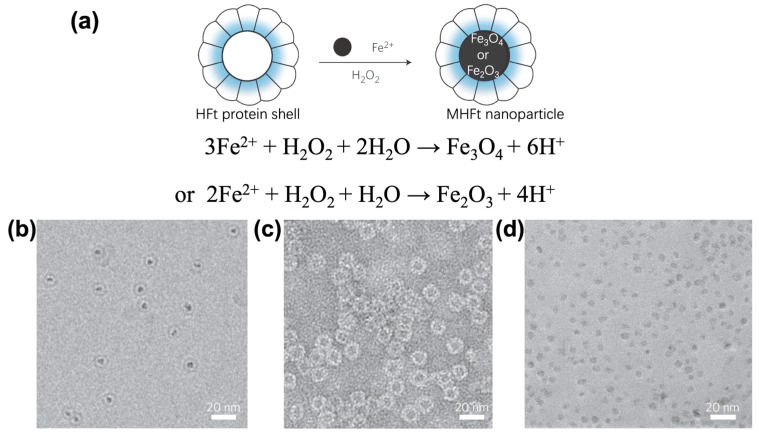
(**a**) The synthesis and structures of magnetoferritin comprised of only H chain subunits; (**b**) cryo-transmission electron microscopy (CryoTEM) image of MHFt nanoparticles; TEM image of (**c**) HFt shell and (**d**) iron core. Reprinted with permission from Ref 41 Copyright 2012 Nature publishing group.

Uchida and co-workers designed a genetically engineered ferritin mutant, comprised of only H chain subunits, thereby avoiding the complex pretreatment of natural ferritin [40]. After prokaryotic expression and purification of recombinant ferritin from *Escherichia coli*, (NH_4_)_2_Fe(SO_4_)·6H_2_O and H_2_O_2_ were simultaneously added to a protein solution under an inert atmosphere, and mineralization was performed at 65 °C and pH 8.5 (Figure 2). The iron core (Fe_3_O_4_ or γ-Fe_2_O_3_) was homogeneously formed due to its different composition of subunits. This MFt allowed for improving the efficiency of biomimetic synthesis, while preserving the biological activity [41,50,51].

Ferritins from bacteria with different properties were also able to form MFt. The *pyrococcus furiosus* (thermostable hyperthermophilic archaeon) ferritin (PfFt) with high stability in extreme high temperature was introduced to the synthesis of MFt by Parker and co-workers [52]. The process of iron oxide mineralization was similar to that of ferritin mentioned above, while the reaction temperature was reach 85 °C. During the process of biomimetic mineralization in vitro, the iron oxide nucleation of PfFt was different from that of mammalian ferritins, which may be because of the different electrostatic potential of the interior PfFt, resulting in significantly lower field strengths of magnetic saturation. Different from the typical octahedral structure of ferritin, *Archaeoglobus fulgidus* ferritin (AfFt) is the only known tetracosameric ferritin forming a tetrahedral cage with four openings. Genetically modified AfFt, AfFt-AA, which could form an octahedral symmetry structure, exhibited a higher stability and a lower release of iron [53]. Amino acid residues Lys-150 and Arg-151 of AfFt were replaced by alanine, which enhanced the hydrophobic interactions of subunits at the 4-fold interface, resulting in the change from tetrahedral to octahedral symmetry. The large triangular pores were eliminated, and the reduction of Fe (III) was possible to be slown down. This AfFt-AA with a high iron loading ability up to 7200 Fe atoms per cage [54].

Other metal ions can be also introduced into the core by doping during the synthesis to change the properties of MFt without changing the particle size [2]. By controlling mixed mineralization reactions of cobalt and iron oxides under mild biomimetic reaction conditions, Fe_3-X_Co_X_O_4_ (X ≤ 0.33) was synthesized in the protein cage of ferritin [55]. A solution of metal ions (Fe^2+^ and Co^2+^ with different proportions) and H_2_O_2_ were added to ferritin solution together to form cobalt-doped MFt [56]. The chemical structure and magnetic properties of these nanoparticles could be tailored by chemical synthesis. 

## 3. Magnetic Properties of Magnetoferritin

The core of natural ferritin consists of iron hydroxide (ferrihydrite) which is antiferromagnetic [57]. In contrast, biomimetic magnetoferritin contains an iron oxide core that consists of superparamagnetic magnetite (or maghemite) [40]. The iron hydroxide core of native ferritin usually consists of 2000–3000 (up to 4500) iron atoms [58]. It has previously been reported that the iron hydroxide core was disordered and non-uniform, which resulted in antiferromagnetic properties [59]. It also reported that small regions of the core can be superparamagnetic, which enable endogenous ferritin serve as an MRI reporter protein [8,60]. However, the lower relaxivity further limited its use for biomedical approaches [61]. 

Compared with native ferritin, MFt containing an iron oxide core (Fe_3_O_4_, γ-Fe_2_O_3_) exhibited superparamagnetic behavior without remanence and coercivity [45]. And synthetic MFt can be prepared in large quantities through bioengineering, maintaining its high biocompatibility [40]. The artificially mineralized iron core exhibits higher blocking temperature, higher sensitivity to be magnetized, and larger anisotropy energy [59]. Different from magnetic iron oxide nanoparticles, magnetostatic interactions were hardly found among MFts due to their intact protein shells [62]. Additionally, MFts were well-dispersed, non-interacting, and randomly oriented. The magnetic properties could be tailored by changing the condition of mineralization, such as controlling the loading of iron and doping of other metal ions [2]. The saturation magnetization of the cores increased as the growth of its size, however, because of the limit of the inner diameter of the hollow sphere, the diameter of core was no more than 8–9 nm [63].

MFt is known as a superparamagnetic protein due to its iron oxide core, however, the magnetic particles inside the cavity are of various sizes and types due to differences in synthesis strategy [2]. The magnetic particles inside the cavity of MFt are generally composed of magnetite (Fe_3_O_4_) or maghemite (γ-Fe_2_O_3_). Magnetic oxide nanoparticles, such as γ-Fe_2_O_3_ generally are disordered, show broken exchange bonds, and a lower surface symmetry when the size decreases, which result in lower saturation magnetization and enhanced magnetic anisotropies [64,65]. The magnetic moment of MFt nanoparticles with γ-Fe_2_O_3_ core was ten times smaller than the crystalline maghemite particles of the same size, because the iron core synthesized inside apoferritin was poorly crystalline and irregular in shape [66]. However, the magnetic anisotropy was much larger and the intensity increases with decreasing size. It was also found that the increase of the loaded iron resulted in a bigger inorganic core and a smaller external diameter of protein. Those magnetic properties were also proved via SQUID (superconducting quantum interference device) and electron magnetic resonance [67]. The iron core could also be controlled as the mixture of hematite (α-Fe_2_O_3_) and maghemite (γ-Fe_2_O_3_) rather than magnetite (Fe_3_O_4_) in appropriate conditions [47]. Additionally, the coercivity and remanence coercivity were larger than those of Fe_3_O_4_ cores due to the hematite cores. At room temperature, this MFt were also superparamagnetic. Under special synthesis conditions, the iron oxide formed inside MFt cores has been exclusively controlled as magnetite (Fe_3_O_4_). In theory, the iron oxide content can be as high as 4500 iron atoms. Atom counting analysis by electron energy-loss spectroscopy (EELS)indicated that the number of Fe atoms/cage was up to 8,400 atoms, which possibly accounted for the high density of magnetite [68]. MFt with Fe_3_O_4_ core are also superparamagnetic. While in cobalt-doped MFt, the exchange bias between antiferromagnetic Co_3_O_4_ and the ferrimagnetic Fe_3_O_4_ core enhanced the magnetic response as a function of temperature. Compared with Fe_3_O_4_ nanoparticles, cobalt ferrite nanoparticles exhibited a higher transverse r2 relaxivity, and particularly enhanced magnetic anisotropy [69,70], a similar phenomenon also appeared on ferritin with cobalt doped. MFt with cobalt-doped core is also superparamagnetic at room temperature. In addition, the MFt can be paramagnetic rather after special modification. The core of tungsten-doped MFt is disordered with un-coupled atomic magnetic moments, resulting in paramagnetic behavior [71]. The paramagnetism could lead to an increased longitudinal relaxivity (r1) and a reduced transverse relaxivity (r2).

Electron diffraction patterns cannot clearly identify the crystals formed in the protein cavity, and Mossbauer spectroscopy requires too large quantities of samples to study the MFt cores. As magnetite and maghemite inside the cavity of MFt generally both showed superparamagnetic behavior, and they have similar diffraction spacings [28]. Several other methods have been used for the detection of crystals. By EELS some structural confusion between magnetite and maghemite can be clarified, and data for the number of Fe atoms was obtained [68]. Micro-Raman spectroscopy is also promising to distinguish iron oxides phases in future studies [72]. Several magnetooptical methods were used to study the iron core of ferritin, and magnetooptical and nuclear magnetic resonance effects might allow to discriminate between a ferrihydrite and a magnetite/maghemite core of ferritin [73]. Furthermore, Faraday rotation spectra and magneto-optical birefringence, which were more economical and convenient than Mossbauer spectroscopy, were introduced to discriminate between maghemite and magnetite cores [74,75].

## 4. Biomedical Applications

MFt has been widely applied in the biomedical field due to its unique properties, including tissue imaging, drug delivery, and medical diagnosis [1]. Artificial MFt has also been used as a model system of pathological ferritin to investigate the corresponding underlying biological mechanism involved [19]. Recently, MFt containing an iron oxide core achieved many advances in biomedical applications, and an additional modification has greatly expanded the field (Table 1).

### 4.1. Magnetic Resonance Imaging

Iron oxide magnetic nanoparticles (MNP) have widely been used for MRI as T1 contrast agents and T2 contrast agents [84]. However, MNPs with great properties require complex modification steps to realize tissue targeting and biocompatibility [85]. MFt also showed superparamagnetic behavior without remanence and coercivity at room temperature [45,86]. MFt was synthesized with the template of ferritin, resulting in uniform size of 12 nm. Compared with MNPs, MFt is well-dispersed and non-interacting in the solution because of the protein shell. The ferritin shell also provides MFt with good biocompatibility, which is suitable for MRI in vivo [38]. The r2 relaxivity of MFt is comparable to known MNP MRI contrast agents [87]. In addition, MFt composed of H chains can target tumor cells without any modification [41], while magnetic properties can be changed by doping of other metal ions [88]. 

In general, MFt acted as T2 MRI agent with good performance [63]. Superparamagnetic iron oxide was synthesized in the cavity of horse spleen apoferritin with high r2/r1 ratio [48]. This MFt provided a good template for modification, which could be used as a contrast agent in MRI. The superparamagnetic core enhanced contrast enhancement for MRI, without cytotoxicity, and induction of reactive oxygen species [89]. MFts were well-dispersed, non-interacting due to its protein shell. Genetically modified ferritin, AfFt-AA, could also be a promising T2 contrast agent [54]. AfFt-AA showed high iron loading ability up to 7200 Fe per cage, and r1 and r2 values were higher than non-modified MFt. This might because the special channel of AfFt-AA could affect the diffusion of water. 

**Figure 3:**
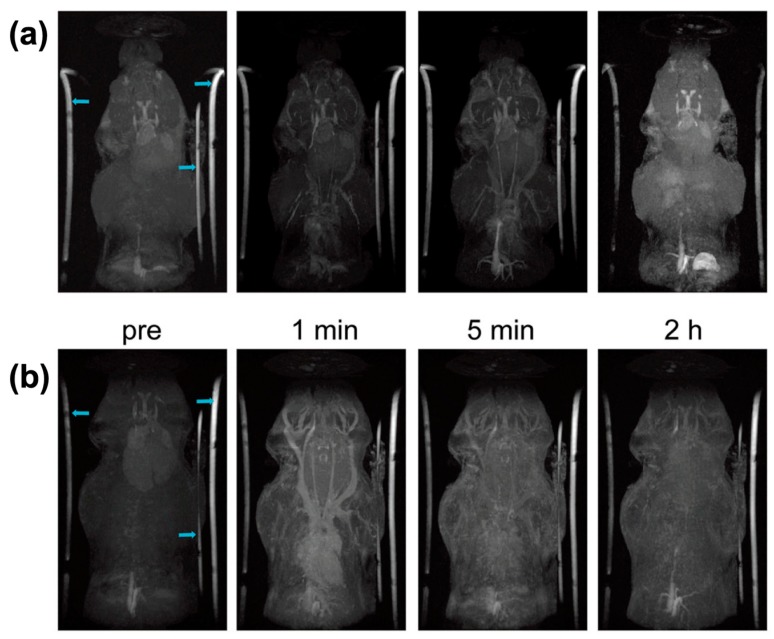
Magnetic resonance angiography of head and neck in mice injected with MFt and Gd-DTPA: (**a**) After injecting MFt, MFt provided continuous contrast enhancements within 2 hours; (**b**) after Gd-DTPA administered, short term blood pool imaging was observed compared with MFt. Reprinted with permission from Ref 47 Copyright 2019 Royal Society of Chemistry.

However, in T2-weighted MRI, the darker contrast might be considered artifacts. Natural ferritin iron and hemosiderin iron could also influence the r2 relaxation rates to generate the T2 MRI signals [90]. However, longitudinal relaxivity (T1) contrast agents can avoid artifacts. T1 agents can interact with local water protons and shorten their spin–lattice relaxation time, thereby resulting in signal brightening [91,92]. A mixed iron oxide with tungsten was successfully deposited inside the ferritin cavity to achieve high T1 relaxivity [71]. After seeding an iron oxide core in apoferritin by the intrinsic ferritin ferroxidase, tungsten and iron were incorporated into the core. The relaxivity could be controlled tungsten addition. The doped tungsten iron that makes it difficult to accept electrons from oxygen bridges could disrupt coupling of atoms by superexchange, leading to formation of an amorphous crystal core. Different from general superparamagnetic MFt, this WFe-apoferritin was paramagnetic. In that case, the values of r2 and r2/r1 were reduced, whereas the surface area for water proton exchange increased. The paramagnetic WFe-apoferritin nanoparticles were detectable in vivo in nano molar concentrations using T1-weighted MRI. The size of iron oxide nanoparticles which used as T1 contrast agents usually less than 5 nm [93]. Through controlled biomimetic mineralization, Cai and co-workers synthesized ultrafine (about 2.2 nm) ferritin-based iron oxide (hematite/maghemite) from genetically recombinant human ferritin [47]. The iron oxide cores were primarily hematite (α-Fe_2_O_3_) or maghemite (γ-Fe_2_O_3_), as determined by Raman spectra, and were superparamagnetic at room temperature. This MFt nanoparticles of 2.2 ±0.7nm had an r1 value of 0.86 mM^−1^ s^−1^ and a r2/r1 ratio of 25.1 that allowed for gaining high spatial resolution steady-state images with long-term magnetic resonance imaging up to 2 hours after single injection of MFt (Figure 3). Furthermore, biodistribution examination also excluded the risk of iron overload. This study was meaningful for the development of a clinical application of Gadolinium free magnetic resonance contrast agents.

### 4.2. Multimodal Imaging

To increase our understanding on tumor diagnosis, in addition to applications in MRI, MFt is flexible to be modified with a functional molecule to realize multimodal imaging, such as positron emission tomography (PET) and fluorescence imaging [1]. Radionuclide 125I was conjugated with human heavy-chain ferritin iron nanocages as a novel MFt nanoprobe (125I-M-HFt), which could internalize into cancer cells via a tumor-specific HFt-TfR1 pathway [80]. Tumor detection in vivo was achieved by both magnetic resonance imaging and SPECT using specific targeting. HFt could bind to human cells via TfR1, which actively recycled independently of ligand binding, thus within a single intravenous injection of 125I-M-HFt highly contrast-enhanced SPECT/MRI tumor imaging was achieved without excessive non-radiolabeled probe blocking. A semigenetic system used a genetic component, which interacted with exogenous components, which realized deep tissue imaging with genetic targetability [94]. It was previously reported that MFt was internalized into cells and subsequent trafficked to lysosomes through semigenetic approaches [79]. By controlling the expression of ferritin receptors, cells could uptake biosynthetic MFt and native ferrihydrite-containing ferritin, which accumulated in lysosomes. This MFt with a magnetite core allowed multimodal and multiscale detection, such as third harmonic generation microscopy (THG), optoacoustic (OA), and MRI. 

Tumors at early stages usually have a low tumor-tonormal ratio, which limits the sensitivity of targeted imaging-based nanoparticles. Nanoparticles use the circulation to be transferred to tumor sites, however, pre-angiogenic tumors are poorly connected to the blood stream, which limits targeting. HFt-based nanoparticles were transported across biological barriers, such as the endothelium and epithelium in vivo [50,81]. HFt labeled with the near-infrared emitting dye Cy5.5 inside the cage could be used in near-infrared fluorescence imaging whereas MHFt was used in MRI [81]. HFt-based nanoparticles could pass through intercellular tight junctions and be endocytosed from clathrincoated pits and caveolae. It is worth noting that MHFt was used to detect brain tumors by MRI, and M-HFt could transport across the blood–brain barrier (BBB) surprisingly. In addition, it was found that BBB endothelial cells (ECs) were proven to overexpress TfR1, and HFt nano-carriers could cross the BBB via receptor-mediated transport without blocking in lysosomes of ECs [50]. The HFt maintained their intact structure and specifically accumulated in brain tumor sites. In tumor cells, HFt seemed to be enriched in lysosomes and degraded to release the stored components. HFt-based nanoparticles show great potential for applications in medical imaging.

### 4.3. Tumor Diagnosis In Vitro

**Figure 4:**
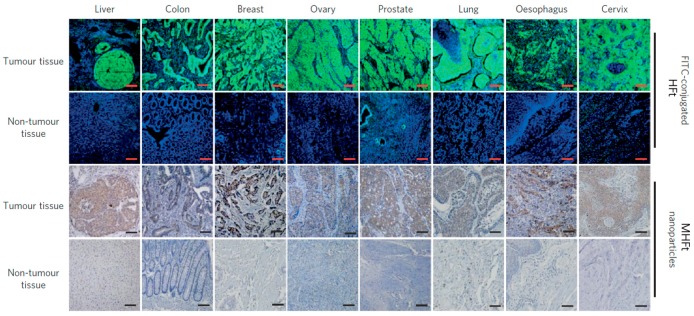
MFt can realize cancer diagnosis via tumor targeting and visualization. Reprinted with permission from Ref 41 Copyright 2012 Nature publishing group.

In the presence of H_2_O_2_, iron oxide nanoparticles, like natural peroxidases, can catalyze the oxidation of peroxidase substrates, that result in a color change [95]. Similarly, MFt nanoparticles were used to target and visualize tumor tissues due to specific targeting of HFt to TfR1 along with peroxidase activity of the iron oxide core [41]. MHFt-based peroxidase staining visualized tumor tissues and provided detailed histopathological information in clinical diagnostics. After reaction of MHFt with cancer cells, H_2_O_2_ and peroxidase substrates were added. Via the hydrophilic channel of MFt, H_2_O_2_ could diffuse into its cavity, and interact with iron oxide that formed OH in situ. Peroxidase substrates nearby were oxidized by OH to generate an insoluble colored precipitate in the position of MFt, which had tumor targeting activity. Staining results of clinical tumor tissue samples realized the visualization of tumors with extreme high sensitivity and specificity (Figure 4). This study provided a fast, economic, and effective tool for cancer detection in the clinic. 

By loading various amounts of iron into recombinant HFt shells, MHFt nanoparticles were synthesized with controlled sizes of magnetite cores in the range of 2.7–5.3 nm. When increasing the size of ferrimagnetic cores, the relaxivity, saturation magnetization, and peroxidase-like activity of MHFt nanoparticles also increased [76]. However, the diameter of the ferritin cavity does not allow bigger Fe_3_O_4_ cores than 8 nm. To increase the peroxidase-like catalytic activity of MHFt, Zhang et al. reported doping of controlled cobalt into the magnetite cores [56]. MHFt of Co_x_Fe_3-x_O_4_ core was synthesized with proportions of cobalt of 0%, 9.0%, 17.2%, and 34.2%, respectively. When doping more cobalt into the magnetite cores, not only the size and shape factor of the cores slightly increased, but also the loop shape, saturation magnetization and coercivity of M-HFt changed like the Cobalt ferrite nanoparticles, as previously reported [96]. Co_3_O_4_ nanoparticles could oxidize TMB directly by Co^3+^ as catalysts [97], and it was found that cobalt-doped M-HFt nanoparticles could improve the catalytic activity, enhancing the enhanced staining efficacy, and maintaining a high targeting ability to tumor tissue. MFt with peroxidase-like catalytic activity made it economic and convenient for the diagnosis of tumor traditional immunohistochemical methods.

Fan et al. reported that nanozymes could be used in tumor therapy [98]. The nitrogen-doped porous carbon nanospheres killed tumor cells by performing their selective activity, with their own peroxidase, oxidase, catalase, and superoxide dismutase activities. In this study, ferritin was introduced to allow for tumor targeting while magnetic properties were lost. Developing MFt in cancer therapy in vivo will maintain the magnetic properties and nanozyme activity and has great potential. 

### 4.4. Therapy of Cancer

In addition to applications, such as tumor imaging and diagnosis, MFt could also be used for tumor therapy as a nanocarrier. RGD-4C, a specific tumor targeting peptide, was genetically incorporated on the exterior surface of HFt, without altering the function of the cage [40]. It enabled HFt to target tumor cells as a nanocarrier. Afterwards, it found that HFt has native tumor targeting [41]. It has been reported that HFt delivered high doses of the tumor drug doxorubicin (Dox) as a nanocarrier, Dox-loaded HFt (HFt-Dox) to realize tumor targeted therapy [51]. MHFt with tumor targeting magnetic proprietary allowed to combine cancer diagnosis and therapy. To target melanoma selectively, α-melanocyte-stimulating hormone (α-MSH) was genetically linked with MFt [78]. The MSH peptide enabled the MFt show more selectivity to target tumor cells. Additionally, the MFt surface was also modified with polyethylene glycol molecules to increase the time of blood circulation. The functional MFt retained the ability to encapsulate magnetic iron core and expected spherical cage-like structures, which was expected to combine MRI agent and nanomedicine carrier in the diagnosis and treatment of melanoma.

**Figure 5:**
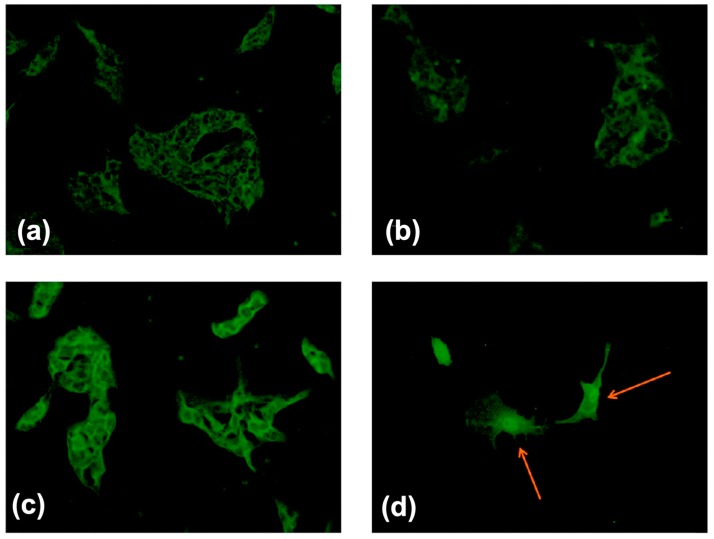
Immunofluorescence analysis of the apoptotic enzyme caspase-3 in B16 cells: (**a**) control cells; (**b**) cells exposed to the magnetic field; (**c**) cells treated with HFt; and (**d**) cells treated with HFt and exposed to the magnetic field. The alternating magnetic field of amplitude H_0_ = 17.0 kA/m and frequency f = 183 kHz was applied for 30 min. Reprinted with permission from Ref 46 Copyright 2014 American Chemical Society.

Radiolabeled biomolecules, such as antibodies and DNAs have been widely applied in radiotherapy and in the detection of tumors. In addition, MFt was labeled with rhenium, which is in +1 oxidation state [82]. Due to the laboratory restrictions on the use of radioactive elements, for the initial study, the non-radioactive rhenium isotope (^187^Re) was used instead of ^188^Re, while the radio rhenium compound was used for radiation therapy. Rhenium labeled MFt with high stability and low toxicity, and was still competent for T2 contrast agents in MRI, while preserving the tumor cell targeting ability. By doping of the core with cobalt, the modified MHFt was allowed hyperthermia for the therapy of tumor under the affection of alternating magnetic field [46] (Figure 5). Enhanced magnetic anisotropy enabled high efficiency of hyperthermia with reduced fields and frequencies. The cobalt doping along with loss of crystallinity resulted in enhanced magnetic anisotropy, which was in conflict with the reported formation of antiferromagnetic Co_3_O_4_ [55]. The semigenetic system, which allowed genetic targetability, enabled magnetic cell manipulation and cell ablation by photoablation or local magnetic hyperthermia [79]. Therefore, combining tumor imaging and tumor therapy is promising for practical applications, and different methods of cancer treatment may benefit from the use of MFt. 

### 4.5. Assembly of Ferritin for Biomedical Applications

Nanoscale ferritin could be assembled into a complex structure to expend biomedical functions in space and time [99]. PfFt with a Fe_3_O_4_-γ-Fe_2_O_3_ iron oxide core and photodegradable Newkome-type dendrons were assembled into micrometer-sized crystal complexes with a face-centered-cubic superstructure by Kostiainen and co-workers [37]. The nanoparticles with fcc superstructures showed different hysteresis of the field-dependent magnetization due to changed magnetostatic interactions. While micrometer-sized crystals were disassembled by a short optical stimulus, free MFt particles regained with typical magnetism of a single domain. The surperstructures allowed the switch between microscale and nanoscale, showing attractive advantages in biomedical applications, such as targeted drug delivery with magnetic control. To enhance relaxivity, a polymer, poly(*N*-methyl-2-vinylpyridinium) iodide-block-poly (ethylene oxide) diblock copolymer was used to mediate the self-assembly of MFt [77]. The self-assembly of MFt was mediated by the polymer, forming stable clusters with a hydrodynamic diameter of 200 nm exhibited lower r1 and higher r2 results in enhanced imaging contrast. Similar to iron oxide nanoparticles, MFt has also been incorporated into cellular spheroids to realize magnetic manipulation, such as fabrication of complex tissues structures [86]. Different from normal magnetic nanoparticles, MFt did not show any side effect on cell viability with long term interaction and high concentration. HFt was also used to fabricate biochemical and magnetic scaffolds [83]. Those micron-sized scaffolds could mimic the microtubule organizing centers of cells and be manipulated via magnetic interactions. In the future, it may be determined that MFt is involved in controlling more biological processes.

### 4.6. Other Bioapplications of Magnetoferritin

MFt could also be used as magnetic marker to magnetize cell. Recently, Carreira and co-workers synthesized a novel cationised MFt that could rapidly magnetize stem cell within one minute and magnetisation was maintained for several weeks [38]. (NH_4_)_2_FeSO_4_·6H_2_O containing CoSO_4_·7H_2_O was oxidized inside the apoferritin cavity by H_2_O_2_ to achieve cobalt-doped MFt (about 1% (*w*/*w*) cobalt doping of the iron oxide). For cationisation, purified MFt was coupled with *N*,*N*-dimethyl-1,3-propanediamine via an amide bond. Cationised MFt with an average particle diameter of 8.2 nm mineralized inside the cavity showed a similar magnetic saturation, susceptibility, and relaxivity with MFt. Due to the electrostatic adsorption of cat-MF to the cell surface, cat-MF realized rapid magnetic labelling, thereby providing lasting MRI contrast and excellent biocompatibility. 

Fully-genetically encoded strategies have enabled to express ferritins in vivo. Magnetoferritin can be synthesized easily in vitro, however it is challenging to be formed genetically in vivo. Ferritin has been attached to an ion channel protein to perform noninvasive magnetic control [100,101]. The magnetic ferritin could be tugged or heated with a magnetic field and the membrane conductance could be controlled, however, it does not obey the basic laws of physics [102,103]. Another magnetic protein, magnetoreceptor (MagR) was proved to be the magnetoreceptor in the body and could form a magnetic protein biocompass with cryptochromes(Cry4) [104]. This biocompass is also contrary to the physical laws [102], however, Cao et al. reported the theoretical possibility of the magnetic moment in MagR [105]. In some pathological tissues, the core of native ferritin might turn from ferrihydrite to magnetite, thereby forming biogenic magnetoferritin [35]. Thus, the expression of magnetoferritin in vivo with good safety is worth being explored for further biomedical applications. Thus, magnetogenetics is an attractive field due to its wonderful prospects.

## 5. Conclusions

Ever since MFt was first synthesized in 1992, it has been widely applied in the biomedical field due to its good biocompatibility, economic accessibility, and unique chemical properties [2]. The synthetic strategies of MFt are flexible and effective, and the biomedical applications of MFt has achieved significant progress. However, the biological function of ferritin and the magnetic properties of the iron core remain to be elucidated. Identifying the biological mechanism of ferritin will help us better understand neurological diseases and iron excess in the body, which will provide novel ideas for biomimetic synthesis and tumor therapy. Though with biocompatibility, biological metabolic pathways and potential side effects of MFt are in urgent need of sufficient research to promote its clinical applications. The shell of MFt can be functionalized by chemical and genetic modifications, whereas the iron core can be modified to gain ideal and unknown properties. It is highly likely that, in the future, MFt will show its charm in tumor imaging, diagnosis, and therapy as a special nanoplatform.

## Figures and Tables

**Table 1:** Magnetoferritin in biomedical applications.

Ferritin Shell	Extra Modification	Properties of Iron Core	Applications	References
apoFt		Superparamagnetic	MRI (T2)	[48]
	W doped	Paramagnetic	MRI (T1)	[71]
HFt		Superparamagnetic	MRI (T2)	[63]
		Peroxidase	Tumor diagnosis	[41,76]
		Superparamagnetic	Native tumor targeting	[41]
	Polymer mediated	Enhanced relaxivity	MRI (T2)	[77]
	RGD linked	Superparamagnetic	Tumor targeting, MRI (T2)	[40]
	α-MSH linked	Superparamagnetic	Selective targeting	[78]
	Ultrafine Fe_2_O_3_	Superparamagnetic	MRI (T1)	[47]
	Co doped	Superparamagnetic, Enhanced relaxivity	Magnetize cells, MRI (T2)	[38]
	Co doped	Peroxidase	Cancer diagnosis	[56]
	Semigenetic	Superparamagnetic	THG, OA and MRI (T2)	[79]
	^125^I conjugated	Superparamagnetic	SPECT imaging, MRI (T2)	[80]
	Co doped	Enhanced magnetic Anisotropy	Hyperthermia	[46]
	Cy5.5 labeled	High relaxivity	NIRF imaging, MRI (T2)	[81]
	Re conjugated	Superparamagnetic	Radiotherapy, MRI (T2)	[82]
	Self-organized	Magnetic scaffold	Control bioprocess	[83]
AfFt-AA		Enhanced relaxivity	MRI (T2)	[54]
PfFt	Assembly	Superparamagnetic	Control drug delivery	[37]

## Abbreviations

MFt	Magnetoferritin
HFt	H chain ferritin
apoFt	Apoferritin
MRI	Magnetic resonance imaging
MHFt	Magnetic HFt
TfR1	Transferrin receptor 1
PfFt	Hyperthermophilic archaeon P. furiosus
AfFt	Archaeoglobus fulgidus ferritin
EELS	Electron energy-loss spectroscopy
SPECT	Single-Photon Emission Computed Tomography
SQUID	Superconducting quantum interference device
THG	Harmonic generation microscopy
OA	Optoacoustic
MNP	Magnetic nanoparticle
NIRF	Near-infrared fluorescence
α-MSH	α-melanocyte-stimulating hormone
BBB	Blood–brain barrier
ECs	Endothelial cells
Dox	Doxorubicin
ROS	Reactive Oxygen Species
